# Associations between modifiable risk factors and cognitive function in middle-aged and older Chinese adults: joint modelling of longitudinal and survival data

**DOI:** 10.3389/fpubh.2024.1485556

**Published:** 2024-11-18

**Authors:** Qin Ran, Fang Yang, Qin Su, Peng Li, Yaoyue Hu

**Affiliations:** ^1^School of Public Health, Chongqing Medical University, Chongqing, China; ^2^Max Planck Institute for Demographic Research, Rostock, Germany

**Keywords:** modifiable risk factors, cognitive function, mortality, joint model, missing not at random

## Abstract

**Background:**

Stronger associations between modifiable risk factors and cognitive function have been found in younger than older adults. This age pattern may be subject to mortality selection and non-ignorable missingness caused by dropouts due to death, but this remains unclear.

**Methods:**

Longitudinal data from 9,562 adults aged 50 and older from Waves 1–4 (2011–2018) of the China Health and Retirement Longitudinal Study were used. Cognitive function was assessed repeatedly using a battery of cognitive tests. Joint models of longitudinal and survival data were applied to examine the associations of modifiable risk factors with cognitive function and mortality.

**Results:**

Worse cognitive function score was associated with being female (coefficient[*β*] = −1.669, 95% confidence interval [CI]: −1.830, −1.511, *p* < 0.001), low education (*β* = −2.672, 95%CI: −2.813, −2.530, *p* < 0.001), rural residence (*β* = −1.204, 95%CI: −1.329, −1.074, *p* < 0.001), stroke (*β* = −0.451, 95%CI: −0.857, −0.051, *p* = 0.030), probable depression (*β* = −1.084, 95%CI: −1.226, −0.941, *p* < 0.001), and current smoking (*β* = −0.284, 95%CI: −0.437, −0.133, *p* < 0.001); whereas dyslipidaemia (*β* = 0.415, 95% CI: 0.207, 0.626, *p* < 0.001), heart disease (*β* = 0.513, 95% CI: 0.328, 0.698, *p* < 0.001), overweight (*β* = 0.365, 95% CI: 0.224, 0.506, *p* < 0.001) and obesity (*β* = 0.264, 95% CI: 0.048, 0.473, *p* = 0.014) were associated with better cognitive function. These associations changed less than 5% when the longitudinal and survival data were modelled separately. An increase in cognitive function over age was associated with reduced mortality risk (hazard ratio: 0.418, 95%CI: 0.333, 0.537, *p* < 0.001). The association between socioeconomic disadvantage and cognitive function was more evident in women than in men, while the associations of socioeconomic disadvantage and lifestyle with cognitive function increased with age.

**Conclusion:**

Mortality selection and non-ignorable missingness caused by dropouts due to death played a minor role in the associations between modifiable risk factors and cognitive function in middle-aged and older Chinese adults.

## Introduction

As individuals age, cognitive impairment and dementia become increasingly prevalent, reducing quality of life and elevating the risk of disability and mortality (1). Utilizing data from the Health and Retirement Study (HRS) in the United States, one study estimated the prevalence of mild cognitive impairment (MCI) at 22% and dementia at 10% among older adults (2). The prevalence of dementia was comparable in Korea (9.2%) (3) and Japan (9.5%) (4). Although a recent nationwide survey in China reported a lower prevalence of MCI (15.5%) and dementia (6.0%) (5), these rates continue to rise in China due to population aging (5), which will place a heavy burden on the families and on the social and healthcare systems.

According to the Lancet Commission on Dementia Prevention, Intervention, and Care, approximately 40% of worldwide dementias are attributable to modifiable risk factors, updated from 9 in 2017 (i.e., less education, smoking, obesity, depression, physical inactivity, low social contact, hypertension, hearing impairment, and diabetes) to 12 in 2020 (adding excessive alcohol consumption, traumatic brain injury, and air pollution) (1, 6). Emerging evidence also points to dyslipidaemia and cardiovascular disease (CVD) as risk factors and the Mediterranean diet as a protective factor for cognitive impairment and dementia (7, 8). A pooled cohort study showed that both insufficient (≤4 h/night) and excessive (≥10 h/night) sleep duration increased the risk of cognitive decline (9). A cross-sectional study from China with a large nationally representative sample of older adults also identified 9 modifiable risk factors for MCI and dementia, including rural residence, less education, being divorced/widowed or living alone, smoking, hypertension, hyperlipidaemia, diabetes, heart disease, and cerebrovascular disease (5). However, the role and contribution of these modifiable risk factors to cognitive decline may vary by sex, rural–urban residence, and age (10, 11). These disparities may be more pronounced in China compared to other countries, due to large differences in cognitive function, education, access to resources, and lifestyle between men and women, age groups, and rural and urban residents (12, 13).

A recent analysis of the UK Biobank data revealed that the associations between most modifiable risk factors and dementia were stronger in younger adults than in their older counterparts (14). However, individuals with poor cognitive function or who experience rapid cognitive decline are known to have a heightened risk of mortality (15, 16). Therefore, it is possible that older adults with unfavourable risk factors may have worse cognitive function or faster cognitive decline, resulting in an increased mortality risk at a younger age. This leaves those who survive have better cognitive function and be more biologically resilient to adverse effects of the unfavourable risk factors (i.e., mortality selection). Furthermore, it is not feasible to follow up individuals after they have died (i.e., dropout due to death). As the probability of missing data caused by dropout due to death depends on the unobserved values of cognitive function, the data are missing not at random (MNAR, i.e., non-ignorable missingness) (17). The relationships between modifiable risk factors and cognitive function at different ages thus may be biased by mortality selection and dropouts due to death. This, to our knowledge, has not been explicitly accounted for in previous studies.

It is crucial to accurately assess the associations between modifiable risk factors and cognitive decline, as this provides invaluable guidance for strategies to prevent cognitive impairment and dementia. Under MNAR, unbiased estimates of cognitive trajectories could be obtained by modelling the trajectories conditionally on the basis of non-random attrition (i.e., death) (17). Our study therefore investigated (1) how sociodemographic characteristics, lifestyle, and health conditions were associated with trajectories of cognitive function in middle-aged and older Chinese adults when the longitudinal and survival processes were jointly modelled; and (2) how these associations differed by sex, rural–urban residence, and age.

## Methods

### Study design

Data came from the China Health and Retirement Longitudinal Study (CHARLS) with a nationally representative sample of community-dwelling adults aged 45 and older. The details of the data used in this study are available on the website,^1^ and the data can be accessed after registration and downloaded upon approval. The baseline survey was conducted in 2011 (Wave 1), and comprehensive information on socio-demographic characteristics, family, health status and functioning, work, retirement, and healthcare was collected. Three follow-up surveys were carried out in 2013 (Wave 2), 2015 (Wave 3), and 2018 (Wave 4), respectively. Both the survival status and the time of death were recorded at Wave 2; yet only the survival status was recorded at Waves 3–4. Details of CHARLS can be found elsewhere (18). We included participants aged≥50 at Wave 1 who reported no memory problems at Wave 1, had at least one cognitive assessment at Waves 1–4, and had no missing data on lifestyle, body mass index (BMI), and health conditions at Wave 1 (*N* = 9,562, Supplementary Figure S1).

### Cognitive function

Cognitive function, consisting of episodic memory and mental status, was assessed at Waves 1–4. After the interviewer read out 10 unrelated words, participants were asked to recall as many words as possible immediately (immediate recall) and approximately five minutes later (delayed recall). One point was awarded for each correctly recalled word. Episodic memory (scored 0–10) was calculated as the average of the immediate word recall (0–10) and delayed word recall (0–10). Mental status (scored 0–11) was captured by time orientation, calculation, and visuospatial ability. Time orientation (scored 0–5) was evaluated by asking participants to name today’s date (month, day, year, and season) and the day of the week. Calculation (scored 0–5) was assessed using the serial 7 s test, which required participants to subtract 7 from 100 up to five times. Visuospatial ability (scored 0–1) was measured by whether participants could re-draw two overlapping pentagons that had been previously shown to them. Cognitive function (scored 0–21) was calculated by summing the scores of episodic memory and mental status, with a higher score indicating better cognitive function (19). In our study, 791, 1,886, 2,792 and 4,524 participants had cognitive function data missing at Wave 1–4, respectively.

### Socio-demographic characteristics

Marital status (married/partnered or unmarried/divorced/widowed), education (<secondary school or ≥ secondary school), and place of residence (rural or urban), were measured at Wave 1. Following the analysis of UK Biobank data (14), education below secondary school and living in rural areas were used to reflect socioeconomic disadvantage, as attaining secondary or higher education and urban residence were strongly correlated with better opportunities, employment, access to resources, and the accumulation of material over the life course for older Chinese adults. One point was assigned for each indicator, and the risk score for socioeconomic disadvantage was calculated as the sum of the two indicators (scored 0–2).

### Lifestyle

Smoking (never/former or current), alcohol drinking in the past year (yes or no), and sleep duration were assessed at Wave 1. Unfavourable sleep duration was defined as ≤6 or ≥ 10 h/night for participants aged 50–64 and ≤ 6 or ≥ 9 h/night for those aged 65 and above (14). The three lifestyle factors scored 0–3. Since CHARLS did not assess participants’ diets and only measured physical activity in half of the sample, we included BMI as a proxy which was categorized into normal weight (<24.0 kg/m^2^), overweight (24.0–27.9 kg/m^2^), and obesity (≥28.0 kg/m^2^) using the cutoffs in the Chinese population (20).

### Health conditions

Health conditions were measured at Wave 1, covering self-reported doctor diagnoses of hypertension, dyslipidaemia, diabetes, heart disease, and stroke, as well as probable depression assessed using the 10-item Centre for Epidemiologic Studies Depression Scale (CES-D-10) with a score ≥ 12 (21). Health conditions therefore scored 0–6. Coding of variables and risk scores is provided in Supplementary Table S1.

### Statistical analyses

The associations between modifiable risk factors, cognitive function, and mortality were analysed using the joint model, which typically combines a linear mixed-effects (LME) model for the longitudinal outcome (i.e., longitudinal sub-model) with a survival model for the survival outcome (i.e., survival sub-model) in a single statistical framework (22). The joint model enables the utilization of the survival outcome to inform trajectories of the longitudinal outcome on dropouts due to death, and vice versa, associate the longitudinal outcome with survival (23). As a result, it corrects trajectories of the longitudinal outcome for non-ignorable missingness caused by dropouts due to death (17) and minimizes the mortality selection bias (23, 24).

We first modelled the trajectories of cognitive function using LME model with age as the time scale (centred at 50). Several LME models were compared including (1) random intercept; (2) random intercept + random slope of age; and (3) random intercept + random slopes of age and age squared. The second model was selected as it had the best model fit (Supplementary Table S2). For the survival outcome, since the exact date of death was unknown (i.e., interval censored), we used the parametric survival model with Weibull distribution (see Supplementary Figure S2; Supplementary Table S3 for the selection of survival distribution) and age as the time scale (25).

In the standard joint model, the two sub-models are connected via the true and unobserved longitudinal outcome at each time point (i.e., “current value” association), which assumes that the hazard of death at time *t* is associated with cognitive function at time *t* (22). There could be other association structures, such as “current slope” (i.e., the hazard of death at time *t* is associated with the slope of the trajectory of cognitive function at *t*), “current value and current slope,” and “time-dependent slope” (i.e., the hazard of death at time *t* is associated with the change of the cognitive function between *t*-1 and *t*, see Supplementary Method) (26). We estimated joint models with these association structures using the Bayesian approach with a Markov Chain Monte Carlo (MCMC) estimation of 20,000 iterations and a burn-in phase of 2000 iterations. Due to non-convergence, age was further divided by 10. The “time-dependent slope” association structure was selected because it had the smallest deviance information criterion (DIC, Supplementary Table S4) and good trace plot and density estimation plot (Supplementary Figure S3) (26). Sociodemographic factors, lifestyle, and health conditions were entered into the joint model both as separate variables and as continuous risk scores. The sample was further stratified by sex, place of residence, and age to examine potential effect modifications. All tests were 2-sided with an *α*-level of 0.05. Statistical analyses were performed in R 4.2.0 (R Core Team, 2022) using the packages of “nlme,” “survival,” and “JMbayes2”.

## Results

In the total of 9,652 participants, 974 (10.2%) died during the follow-up (Table 1). Majority of the participants were married/partnered, had received primary school or lower education, and were living in rural areas. The proportion of low education was higher among older individuals, women, and rural residents. The most prevalent health conditions were probable depression (28.8%) and hypertension (26.2%). Smoking, alcohol drinking, and overweight and obesity were more prevalent in men than in women, while more than half of participants had an unfavourable sleep duration.

**Table 1:** Sample characteristics (*N* = 9,562).

	Total sample (*N* = 9,562)	Age (years)			Sex		Place of residence	
		50–59 (*N* = 4,456)	60–69 (*N* = 3,444)	≥70 (*N* = 1,662)	Men (*N* = 4,713)	Women (*N* = 4,849)	Urban (*N* = 3,529)	Rural (*N* = 6,033)
Death (%)	974 (10.2)	185 (4.2)	352 (10.2)	437 (26.3)	619 (13.1)	355 (7.3)	335 (9.5)	639 (10.6)
Age (mean, SD)	62.2 (7.9)	55.5 (2.7)	64.4 (2.9)	75.4 (4.4)	62.3 (7.8)	62.0 (8.0)	62.3 (8.0)	62.1 (7.8)
Cognitive function (mean, SD)								
Wave 1	10.9 (4.0)	11.6 (3.8)	11.0 (3.9)	8.9 (4.2)	11.9 (3.5)	9.9 (4.3)	12.1 (3.8)	10.2 (4.0)
Wave 2	11.6 (3.6)	12.0 (3.4)	11.6 (3.5)	10.0 (3.9)	12.2 (3.2)	10.9 (3.8)	12.6 (3.4)	11.0 (3.6)
Wave 3	10.2 (4.4)	11.0 (4.1)	10.1 (4.3)	7.7 (4.5)	11.3 (3.7)	9.0 (4.6)	11.4 (4.1)	9.5 (4.3)
Wave 4	10.6 (4.3)	11.0 (4.2)	10.5 (4.2)	8.6 (4.6)	11.4 (3.8)	9.8 (4.7)	11.9 (4.0)	9.9 (4.3)
Sex (%)								
Men	4,713 (49.3)	2,123 (47.6)	1749 (50.8)	841 (50.6)			1,681 (47.6)	3,032 (50.3)
Women	4,849 (50.7)	2,333 (52.4)	1,695 (49.2)	821 (49.4)			1848 (52.4)	3,001 (49.7)
Marital status (%)								
Married/partnered	8,199 (85.7)	4,147 (93.1)	2,957 (85.9)	1,094 (65.9)	4,238 (89.9)	3,961 (81.7)	3,026 (85.7)	5,173 (85.7)
Unmarried/divorced/widowed	1,363 (14.3)	309 (6.9)	487 (14.1)	567 (34.1)	475 (10.1)	888 (18.3)	503 (14.3)	860 (14.3)
Socioeconomic disadvantage (%)								
Low education	7,016 (73.4)	2,844 (63.8)	2,721 (79.0)	1,451 (87.3)	3,023 (64.1)	3,993 (82.3)	2,148 (60.9)	4,868 (80.7)
Rising in rural areas	6,033 (63.1)	2,820 (63.3)	2,210 (64.2)	1,003 (60.3)	3,032 (64.3)	3,001 (61.9)		
Health conditions (%)								
Hypertension	2,507 (26.2)	926 (20.8)	1,045 (30.3)	536 (32.3)	1,113 (23.6)	1,394 (28.7)	1,066 (30.2)	1,441 (23.9)
Diabetes	645 (6.7)	260 (5.8)	283 (8.2)	102 (6.1)	259 (5.5)	386 (8.0)	354 (10.0)	291 (4.8)
Dyslipidaemia	955 (10.0)	452 (10.1)	385 (11.2)	118 (7.1)	402 (8.5)	553 (11.4)	498 (14.1)	457 (7.6)
Heart disease	1,248 (13.1)	446 (10.0)	508 (14.8)	294 (17.7)	530 (11.2)	718 (14.8)	606 (17.2)	642 (10.6)
Stroke	240 (2.5)	75 (1.7)	109 (3.2)	56 (3.4)	123 (2.6)	117 (2.4)	94 (2.7)	146 (2.4)
Probable depression^a^	2,752 (28.8)	1,192 (26.8)	1,038 (30.1)	522 (31.4)	1,042 (22.1)	1710 (35.3)	782 (22.2)	1970 (32.7)
Lifestyle (%)								
Current smoking	3,059 (32.0)	1,467 (32.9)	1,119 (32.5)	473 (28.5)	2,730 (57.9)	329 (6.8)	1,008 (28.6)	2051 (34.0)
Current drink	3,135 (32.8)	1,560 (35.0)	1,114 (32.3)	461 (27.7)	2,570 (54.5)	565 (11.7)	1,106 (31.3)	2029 (33.6)
Unfavourable sleep duration^b^	5,549 (58.0)	2,413 (54.2)	2046 (59.4)	1,090 (65.6)	2,605 (55.3)	2,944 (60.7)	1980 (56.1)	3,569 (59.2)
BMI (kg/m^2^, %)								
<24.0	5,912 (61.8)	2,600 (58.3)	2,101 (61.0)	1,211 (72.9)	3,231 (68.6)	2,681 (55.3)	1840 (52.1)	4,072 (67.5)
24.0–27.9	2,657 (27.8)	1,334 (29.9)	991 (28.8)	332 (20.0)	1,153 (24.5)	1,504 (31.0)	1,186 (33.6)	1,471 (24.4)
≥28.0	993 (10.4)	522 (11.7)	352 (10.2)	119 (7.2)	329 (7.0)	664 (13.7)	503 (14.3)	490 (8.1)

SD: standard deviation; BMI: body mass index. Age, sex, marital status, socioeconomic disadvantage, health conditions, lifestyle and BMI were assessed at Wave1.

a CES-D-10 ≥ 12.

b Sleep duration ≤ 6 or ≥ 10 h/night for age 50–64, ≤6 or ≥ 9 h/night for age ≥ 65.

The decline in cognitive function accelerated with age, which was slightly more rapid in the joint model than in the LME model (see Table 2 for *β* coefficients, which indicate how much cognitive function changed with one unit increase in independent variables). In the joint model, worse cognitive function was associated with being female (*β*: −1.669, 95% confidence interval [CI]: −1.830, −1.511, *p* < 0.001), low education (*β* = −2.672, 95%CI: −2.813, −2.530, *p* < 0.001), rural residence (*β* = −1.204, 95%CI: −1.329, −1.074, *p* < 0.001), stroke (*β* = −0.451, 95%CI: −0.857, −0.051, *p* = 0.030), probable depression (*β* = −1.084, 95%CI: −1.226, −0.941, *p* < 0.001), and current smoking (*β* = −0.284, 95%CI: −0.437, −0.133, *p* < 0.001). These associations estimated in the LME model were very similar to those in the joint model (differences<1% but 2.8% for current smoking). In addition, dyslipidaemia (*β* = 0.415, 95%CI: 0.207, 0.626, *p* < 0.001), heart disease (*β* = 0.513, 95%CI: 0.328, 0.698, *p* < 0.001), overweight (*β* = 0.365, 95%CI: 0.224, 0.506, *p* < 0.001), and obesity (*β* = 0.264, 95%CI: 0.048, 0.473, *p* = 0.014) were associated with better cognitive function in the joint model, but these associations were 1.9–4.9% larger in the LME model except for heart disease. For the survival process, in the joint model, diabetes, heart disease, stroke, probable depression, and current smoking were associated with a higher mortality risk. These associations were attenuated by 6.9–11.1% in the parametric survival model, except for stroke (attenuation of 1.5%). The time-dependent slope showed that the mortality risk was reduced by almost 60% (hazard ratio [HR] = 0.418, 95%CI: 0.333, 0.537, *p* < 0.001) with one unit increase in cognitive function over one unit increase over age (i.e., 10 years).

**Table 2:** Associations of modifiable risk factors with cognitive function and mortality.

	Separate model		Joint model	
	β/HR (95% CI)	*p*	β/HR (95% CI)	*p*
**Longitudinal process**				
Age	0.045 (−0.161, 0.251)	0.668	0.072 (−0.121, 0.198)	0.471
Age squared	−0.453 (−0.516, −0.390)	<0.001^***^	−0.484 (−0.530, −0.425)	<0.001^***^
Women	−1.665 (−1.827, −1.504)	<0.001^***^	−1.669 (−1.830, −1.511)	<0.001^***^
Unmarried/divorced/widowed	−0.171 (−0.364, 0.021)	0.080	−0.129 (−0.316, 0.061)	0.185
Socioeconomic disadvantage				
Low education	−2.693 (−2.838, −2.548)	<0.001^***^	−2.672 (−2.813, −2.530)	<0.001^***^
Residing in rural areas	−1.199 (−1.331, −1.068)	<0.001^***^	−1.204 (−1.329, −1.074)	<0.001^***^
Health conditions				
Hypertension	0.126 (−0.025, 0.277)	0.102	0.135 (−0.019, 0.286)	0.084
Diabetes	0.113 (−0.141, 0.367)	0.384	0.110 (−0.141, 0.358)	0.377
Dyslipidaemia	0.423 (0.208, 0.638)	<0.001^***^	0.415 (0.207, 0.626)	<0.001^***^
Heart disease	0.508 (0.316, 0.699)	<0.001^***^	0.513 (0.328, 0.698)	<0.001^***^
Stroke	−0.451 (−0.860, −0.043)	0.030^*^	−0.451 (−0.857, −0.051)	0.030^*^
Probable depression^a^	−1.076 (−1.218, −0.935)	<0.001^***^	−1.084 (−1.226, −0.941)	<0.001^***^
Lifestyle				
Current smoking	−0.276 (−0.436, −0.117)	<0.001^***^	−0.284 (−0.437, −0.133)	<0.001^***^
Current drink	−0.049 (−0.196, 0.099)	0.516	−0.052 (−0.199, 0.092)	0.483
Unfavourable sleep duration^b^	−0.120 (−0.245, 0.005)	0.059	−0.116 (−0.240, 0.004)	0.060
BMI (ref: <24.0 kg/m^2^)				
24.0–27.9 kg/m^2^	0.380 (0.236, 0.524)	<0.001^***^	0.365 (0.224, 0.506)	<0.001^***^
≥28.0 kg/m^2^	0.277 (0.063, 0.490)	0.011^*^	0.264 (0.048, 0.473)	0.014^*^
**Survival process**				
Women	0.595 (0.507, 0.698)	<0.001^***^	0.558 (0.448, 0.691)	<0.001^***^
Unmarried/divorced/widowed	0.930 (0.798, 1.085)	0.355	0.855 (0.721, 1.011)	0.067
Socioeconomic disadvantage				
Low education	0.980 (0.821, 1.169)	0.821	0.936 (0.786, 1.121)	0.469
Residing in rural area	1.136 (0.987, 1.307)	0.076	1.138 (0.992, 1.311)	0.065
Health conditions				
Hypertension	1.112 (0.965, 1.282)	0.143	1.100 (0.955, 1.264)	0.188
Diabetes	1.524 (1.210, 1.920)	<0.001^***^	1.563 (1.223, 1.962)	<0.001^***^
Dyslipidaemia	1.032 (0.814, 1.308)	0.795	1.052 (0.826, 1.325)	0.675
Heart disease	1.236 (1.041, 1.467)	0.015^*^	1.258 (1.056, 1.492)	0.009^**^
Stroke	1.406 (1.051, 1.880)	0.022^*^	1.400 (1.034, 1.867)	0.029^*^
Probable depression^a^	1.255 (1.091, 1.443)	0.001^**^	1.281 (1.110, 1.473)	<0.001^***^
Lifestyle				
Current smoking	1.249 (1.078, 1.448)	0.003^**^	1.280 (1.096, 1.493)	0.003 ^**^
Current drink	1.018 (0.881, 1.176)	0.809	1.002 (0.862, 1.160)	0.978
Unfavourable sleep duration^b^	0.883 (0.774, 1.008)	0.064	0.877 (0.768, 1.001)	0.051
BMI (ref: <24.0 kg/m^2^)				
24.0–27.9 kg/m^2^	0.989 (0.841, 1.163)	0.889	1.023 (0.869, 1.198)	0.796
≥28.0 kg/m^2^	1.023 (0.796, 1.316)	0.857	1.046 (0.808, 1.349)	0.722
Time-dependent slope			0.418 (0.333, 0.537)	<0.001^***^

a CES-D-10 ≥ 12.

b Sleep duration ≤ 6 or ≥ 10 h/night for age 50–64, ≤6 or ≥ 9 h/night for age ≥ 65.

β: model coefficient; HR: hazard ratio; CI: confidence interval; BMI: body mass index; ref: reference category. *p < 0.05, ** *p* < 0.01, *** *p* < 0.001.

When the sample was stratified by sex, the effects of modifiable risk factors on cognitive function were greater in women than in men, except for marital status and smoking (Table 3). Similarly, larger associations were observed in urban than in rural residents, except for dyslipidaemia and heart disease (Table 3). Stratifying the sample by age group, the associations of being female, low education, rural residence, heart disease, and overweight with cognitive function increased with age (Table 4). Only in the oldest age group was worse cognitive function associated with hypertension and current smoking, whereas better cognitive function associated with dyslipidaemia and obesity was observed only in younger age groups. Although the protective effect of the time-dependent slope on mortality was marginally larger in urban than in rural residents, it was much stronger in women and in the oldest age group.

**Table 3:** Associations of risk factors with cognitive function and survival by sex and residence in the joint models.

	Men		Women		Urban		Rural	
	β/HR (95% CI)	*p*	β/HR (95% CI)	*p*	β/HR (95% CI)	*p*	β/HR (95% CI)	*p*
**Longitudinal process**								
Age	0.171 (−0.099, 0.425)	0.257	−0.126 (−0.396, 0.171)	0.418	0.272 (0.005, 0.537)	0.045^*^	−0.141 (−0.455, 0.100)	0.275
Age squared	−0.462 (−0.544, −0.375)	<0.001^***^	−0.476 (−0.558, −0.405)	<0.001^***^	−0.482 (−0.566, −0.393)	<0.001^***^	−0.457 (−0.532, −0.360)	<0.001^***^
Women					−0.902 (−1.139, −0.664)	<0.001^***^	−2.172 (−2.383, −1.962)	<0.001^***^
Unmarried/divorced/widowed	−0.482 (−0.756, −0.208)	<0.001^***^	0.118 (−0.144, 0.387)	0.375	−0.198 (−0.498, −0.098)	0.185	−0.160 (−0.407, 0.088)	0.211
Socioeconomic disadvantage								
Low education	−2.027 (−2.191, −1.863)	<0.001^***^	−3.686 (−3.936, −3.433)	<0.001^***^	−2.855 (−3.048, −2.662)	<0.001^***^	−2.451 (−2.652, −2.250)	<0.001^***^
Residing in rural areas	−0.790 (−0.957, −0.628)	<0.001^***^	−1.440 (−1.638, −1.245)	<0.001^***^				
Health conditions								
Hypertension	0.138 (−0.060, 0.336)	0.170	0.163 (−0.049, 0.380)	0.136	0.237 (0.017, 0.463)	0.034^*^	0.071 (−0.128, 0.263)	0.479
Diabetes	0.119 (−0.229, 0.466)	0.504	0.066 (−0.287, 0.403)	0.696	0.100 (−0.225, 0.421)	0.543	0.077 (−0.299, 0.448)	0.694
Dyslipidaemia	0.356 (0.063, 0.640)	0.017^*^	0.441 (0.138, 0.739)	0.003^**^	0.325 (0.047, 0.607)	0.022^*^	0.471 (0.162, 0.779)	0.004^**^
Heart disease	0.370 (0.106, 0.635)	0.007^**^	0.574 (0.302, 0.843)	<0.001^***^	0.371 (0.112, 0.637)	0.006^**^	0.587 (0.332, 0.847)	<0.001^***^
Stroke	−0.109 (−0.634, 0.409)	0.679	−0.885 (−1.497, −0.258)	0.004^**^	−0.667 (−1.260, −0.060)	0.031^*^	−0.366 (−0.890, 0.150)	0.171
Probable depression^a^	−1.038 (−1.230, −0.840)	<0.001^***^	−1.069 (−1.265, −0.872)	<0.001^***^	−1.267 (−1.499, −1.038)	<0.001^***^	−0.986 (−1.138, −0.798)	<0.001^***^
Lifestyle								
Current smoking	−0.422 (−0.583, −0.262)	<0.001^***^	0.226 (−0.133, 0.587)	0.225	−0.371 (−0.607, −0.129)	0.003^**^	−0.292 (−0.490, −0.088)	0.005^**^
Current drink	0.002 (−0.154, 0.163)	0.985	−0.048 (−0.338, 0.235)	0.756	0.138 (−0.085, 0.359)	0.225	−0.147 (−0.334, 0.041)	0.126
Unfavourable sleep duration^b^	−0.071 (−0.221, 0.084)	0.359	−0.136 (−0.323, 0.051)	0.143	−0.150 (−0.336, 0.038)	0.120	−0.080 (−0.241, 0.077)	0.326
BMI (ref: <24.0 kg/m^2^)								
24.0–27.9 kg/m^2^	0.242 (0.051, 0.428)	0.013^*^	0.550 (0.347, 0.757)	<0.001^***^	0.495 (0.285, 0.705)	<0.001^***^	0.301 (0.113, 0.489)	0.001 ^**^
≥28.0 kg/m^2^	0.270 (−0.038, 0.580)	0.085	0.289 (−0.001, 0.576)	0.051	0.249 (−0.040, 0.534)	0.093	0.274 (−0.023, 0.568)	0.070
**Survival process**								
Women					0.504 (0.349, 0.732)	<0.001^***^	0.574 (0.448, 0.742)	<0.001^***^
Unmarried/divorced/widowed	0.869 (0.653, 1.152)	0.349	0.815 (0.596, 1.101)	0.189	0.900 (0.656, 1.230)	0.515	0.832 (0.678, 1.017)	0.072
Socioeconomic disadvantage								
Low education	0.987 (0.798, 1.230)	0.915	0.715 (0.478, 1.088)	0.110	0.909 (0.700, 1.178)	0.470	0.914 (0.710, 1.184)	0.493
Residing in rural areas	1.073 (0.898, 1.283)	0.445	1.296 (1.028, 1.644)	0.026^*^				
Health conditions								
Hypertension	1.168 (0.978, 1.392)	0.094	1.001 (0.795, 1.263)	0.999	1.136 (0.894, 1.438)	0.298	1.075 (0.899, 1.281)	0.423
Diabetes	1.186 (0.848, 1.628)	0.306	2.125 (1.500, 2.965)	<0.001^***^	1.313 (0.925, 1.833)	0.126	1.964 (1.408, 2.700)	<0.001^***^
Dyslipidaemia	1.001 (0.725, 1.359)	0.986	1.096 (0.753, 1.579)	0.621	1.097 (0.776, 1.523)	0.581	1.001 (0.705, 1.388)	0.979
Heart disease	1.183 (0.937, 1.481)	0.153	1.378 (1.034, 1.801)	0.031^*^	1.148 (0.867, 1.508)	0.317	1.389 (1.102, 1.742)	0.554
Stroke	1.240 (0.842, 1.776)	0.262	1.709 (1.005, 2.757)	0.048^*^	1.727 (1.075, 2.696)	0.025^*^	1.238 (0.824, 1.797)	0.293
Probable depression^a^	1.208 (1.002, 1.452)	0.047^*^	1.387 (1.111, 1.736)	0.004^**^	1.541 (1.186, 1.983)	<0.001^***^	1.170 (0.991, 1.390)	0.066
Lifestyle								
Current smoking	1.210 (1.029, 1.418)	0.022^*^	1.513 (1.065, 2.088)	0.019^*^	1.359 (1.039, 1.763)	0.025^*^	1.238 (1.022, 1.497)	0.026^*^
Current drink	0.965 (0.822, 1.130)	0.662	1.168 (0.841, 1.602)	0.335	1.041 (0.800, 1.346)	0.759	0.988 (0.828, 1.189)	0.895
Unfavourable sleep duration^b^	0.930 (0.791, 1.101)	0.390	0.792 (0.633, 0.989)	0.039^*^	0.770 (0.613, 0.965)	0.025^*^	0.937 (0.796, 1.108)	0.442
BMI (ref: <24.0 kg/m^2^)								
24.0–27.9 kg/m^2^	1.019 (0.817, 1.255)	0.851	1.026 (0.785, 1.323)	0.836	0.964 (0.740, 1.249)	0.784	1.034 (0.834, 1.278)	0.746
≥28.0 kg/m^2^	1.252 (0.880, 1.738)	0.202	0.896 (0.607, 1.288)	0.576	0.980 (0.665, 1.426)	0.926	1.048 (0.734, 1.467)	0.779
Time-dependent slope	0.562 (0.452, 0.706)	<0.001^***^	0.221 (0.134, 0.350)	<0.001^***^	0.354 (0.242, 0.500)	<0.001^***^	0.411 (0.293, 0.571)	<0.001^***^

a CES-D-10 ≥ 12.

b Sleep duration ≤ 6 or ≥ 10 h/night for age 50–64, ≤6 or ≥ 9 h/night for age ≥ 65.

β: model coefficient; HR: hazard ratio; CI: confidence interval; BMI: body mass index; ref: reference category. *p < 0.05, ** *p* < 0.01, *** *p* < 0.001.

**Table 4:** Associations of risk factors with cognitive function and survival by age group in the joint models.

	50–59 years		60–69 years		≥70 years	
	β/HR (95% CI)	*p*	β/HR (95% CI)	*p*	β/HR (95% CI)	*p*
**Longitudinal process**						
Age	0.460 (0.018, 0.886)	0.034*	−0.643 (−1.264, −0.096)	0.022*	−2.853 (−3.409, −2.197)	<0.001***
Age squared	−1.088 (−1.333, −0.830)	<0.001***	−1.004 (−1.357, −0.627)	<0.001***	−0.189 (−0.501, 0.176)	0.351
Women	−1.061 (−1.298, −0.829)	<0.001***	−1.774 (−2.033, −1.514)	<0.001***	−2.589 (−2.956, −2.219)	<0.001***
Unmarried/divorced/widowed	−0.361 (−0.697, −0.025)	0.034*	−0.271 (−0.571, 0.026)	0.074	0.131 (−0.210, 0.475)	0.445
Socioeconomic disadvantage						
Low education	−2.726 (−2.911, −2.543)	<0.001***	−2.969 (−3.240, −2.696)	<0.001***	−3.174 (−3.651, −2.701)	<0.001***
Residing in rural areas	−1.141 (−1.321, −0.959)	<0.001***	−1.079 (−1.303, −0.860)	<0.001***	−1.315 (−1.646, −0.983)	<0.001***
Health conditions						
Hypertension	0.130 (−0.095, 0.352)	0.261	0.114 (−0.125, 0.356)	0.352	−0.348 (−0.683, −0.004)	0.047*
Diabetes	−0.073 (−0.434, 0.295)	0.698	0.222 (−0.153, 0.600)	0.246	0.022 (−0.632, 0.677)	0.952
Dyslipidaemia	0.414 (0.122, 0.708)	0.004**	0.416 (0.067, 0.757)	0.019*	0.603 (−0.018, 1.221)	0.056
Heart disease	0.430 (0.146, 0.720)	0.003**	0.200 (−0.104, 0.501)	0.202	0.710 (0.285, 1.142)	0.001**
Stroke	−0.588 (−1.251, 0.074)	0.082	−0.302 (−0.913, 0.308)	0.329	−0.815 (−1.677, 0.052)	0.064
Probable depression^a^	−1.152 (−1.346, −0.954)	<0.001***	−0.952 (−1.183, −0.719)	<0.001***	−1.234 (−1.575, −0.895)	<0.001***
Lifestyle						
Current smoking	−0.079 (−0.302, 0.148)	0.492	−0.094 (−0.359, 0.162)	0.477	−0.711 (−1.085, −0.335)	<0.001***
Current drink	0.036 (−0.168, 0.239)	0.725	0.062 (−0.179, 0.312)	0.629	−0.180 (−0.548, 0.182)	0.348
Unfavourable sleep duration^b^	−0.188 (−0.356, −0.018)	0.027*	−0.096 (−0.309, 0.109)	0.366	−0.094 (−0.420, 0.236)	0.574
BMI (ref: <24.0 kg/m^2^)						
24.0–27.9 kg/m^2^	0.244 (0.055, 0.432)	0.012*	0.607 (0.367, 0.849)	<0.001***	0.536 (0.130, 0.928)	0.009**
≥28.0 kg/m^2^	0.369 (0.086, 0.646)	0.010*	0.273 (−0.084, 0.633)	0.137	0.195 (−0.408, 0.802)	0.530
**Survival process**						
Women	0.437 (0.263, 0.729)	0.002**	0.435 (0.310, 0.608)	<0.001***	0.445 (0.134, 0.793)	0.001**
Unmarried/divorced/widowed	2.325 (1.479, 3.544)	<0.001***	0.879 (0.620, 1.216)	0.453	0.848 (0.494, 1.246)	0.399
Socioeconomic disadvantage						
Low education	0.825 (0.597, 1.136)	0.240	1.636 (1.229, 2.208)	<0.001***	1.457 (0.848, 3.612)	0.195
Residing in rural areas	0.969 (0.720, 1.323)	0.819	0.887 (0.704, 1.109)	0.295	1.432 (1.033, 2.811)	0.047
Health conditions						
Hypertension	1.490 (1.033, 2.122)	0.033*	1.301 (1.029, 1.640)	0030*	1.160 (0.797, 1.937)	0.403
Diabetes	1.430 (0.777, 2.504)	0.225	1.831 (1.297, 2.541)	0.001**	1.747 (0.875, 6.504)	0.124
Dyslipidaemia	0.909 (0.543, 1.490)	0.727	0.814 (0.564, 1.149)	0.265	0.928 (0.336, 2.057)	0.885
Heart disease	1.163 (0.710, 1.824)	0.529	1.313 (0.980, 1.753)	0.066	2.362 (1.360, 9.289)	<0.001***
Stroke	1.103 (0.390, 2.686)	0.800	2.387 (1.561, 3.526)	<0.001***	1.053 (0.417, 3.157)	0.926
Probable depression^a^	1.268 (0.914, 1.756)	0.158	1.261 (0.991, 1.588)	0.058	1.391 (0.977, 2.778)	0.068
Lifestyle						
Current smoking	1.486 (1.024, 2.193)	0.035^*^	1.031 (0.798, 1.334)	0.820	1.364 (0.928, 2.822)	0.129
Current drink	0.772 (0.549, 1.094)	0.144	1.101 (0.868, 1.402)	0.444	0.856 (0.522, 1.300)	0.392
Unfavourable sleep duration^b^	1.015 (0.755, 1.363)	0.921	0.953 (0.769, 1.188)	0.658	0.737 (0.350, 1.039)	0.096
BMI (ref: <24.0 kg/m^2^)						
24.0–27.9 kg/m^2^	0.718 (0.494, 1.022)	0.066	0.969 (0.747, 1.251)	0.909	0.933 (0.519, 1.466)	0.775
≥28.0 kg/m^2^	0.886 (0.517, 1.452)	0.665	0.987 (0.670, 1.430)	0.961	0.534 (0.158, 1.153)	0.112
Time-dependent slope	0.688 (0.544, 0.879)	0.002^**^	0.716 (0.612, 0.832)	<0.001^***^	0.034 (0.001, 0.349)	<0.001^***^

a CES-D-10 ≥ 12.

b Sleep duration ≤ 6 or ≥ 10 h/night for age 50–64, ≤6 or ≥ 9 h/night for age ≥ 65.

β: model coefficient; HR: hazard ratio; CI: confidence interval; BMI: body mass index; ref: reference category. *p < 0.05, ** *p* < 0.01, *** *p* < 0.001.

**Figure 1:**
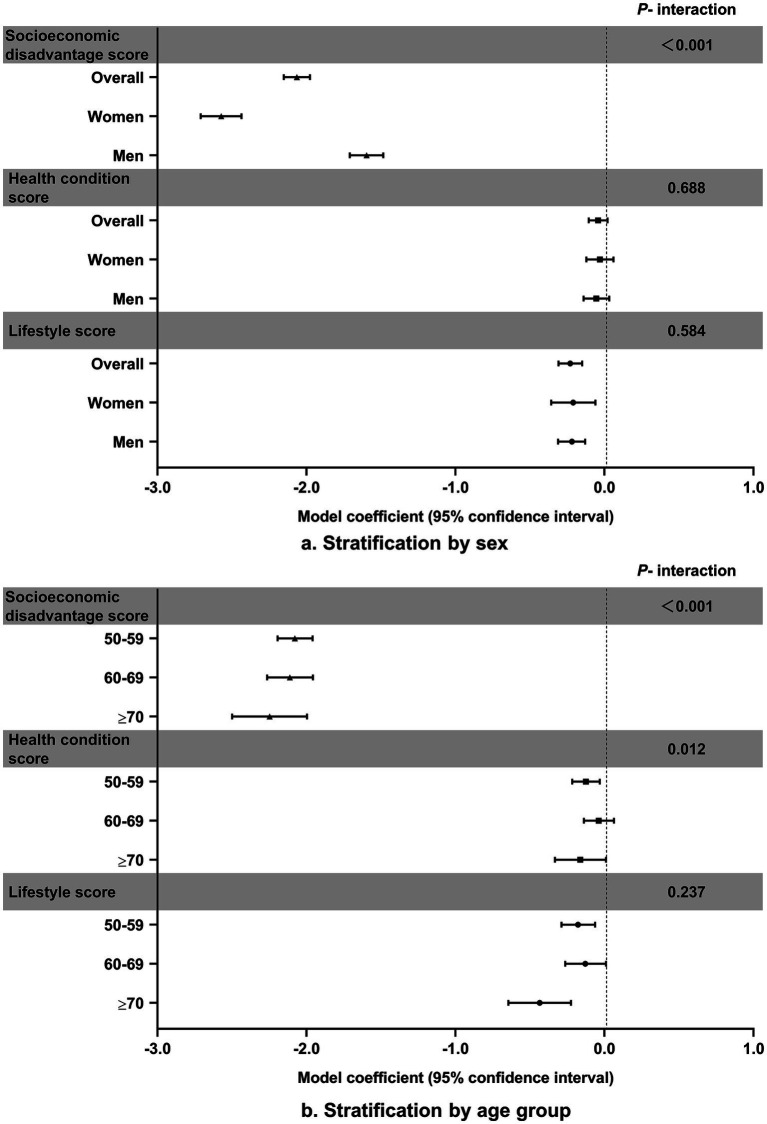
Associations of risk scores for socioeconomic disadvantage, lifestyle, and health conditions with cognitive function.

Figure 1 depicts the associations of the risk scores of socioeconomic disadvantage, lifestyle, and health conditions with cognitive function by sex and age in the joint model (Supplementary Table S5). There were no sex differences in the associations of lifestyle and health conditions with cognitive function, but the adverse effect of socioeconomic disadvantage was much greater in women than in men. Furthermore, the effects of socioeconomic disadvantage and lifestyle on cognitive function were more pronounced at older ages.

## Discussion

In this study of 9,562 Chinese adults aged 50 and over with 7 years of follow-up, socioeconomic disadvantage, lifestyle, and health conditions were associated with trajectories of cognitive function. Joint modelling of the longitudinal and survival processes produced minor changes in these associations compared to when modelling separately. The detrimental effect of socioeconomic disadvantage on cognitive function differed substantially by sex. An age pattern was found in the associations between socioeconomic disadvantage, lifestyle, and cognitive function, but not for health conditions including hypertension, dyslipidaemia, diabetes, heart disease, stroke, and probable depression.

Consistent with a prior study from China (5), we found that lower education and rural residence were associated with worse cognitive function. Longer education may enhance cognitive reserve, providing a buffer against dementia-related brain pathology (27). Inadequate healthcare, less infrastructure, and higher rates of comorbidity in rural China may exacerbate rural–urban disparities in cognitive function, as well as in the associations between modifiable risk factors and cognitive function (11). In our subgroup analysis, women and older age groups showed stronger associations between socioeconomic disadvantage and cognitive function. These findings mainly relate to their lower levels of education. Numerous previous studies on health inequality and the social determinants of health have consistently confirmed that socioeconomic status (SES) – mainly reflected by education, occupation, and income – has profound impacts on health throughout the lifespan (28, 29). Building on these findings, researchers have developed and tested theories of how education influences cognitive function via occupation, income, access to resources, and material accumulation (30, 31). A population-based cohort study of 7,357 Americans aged 45 and older showed that occupational complexity mediated 11–22% of the protective effect of education on cognitive function (32). Moreover, another large-scale survey of adults aged 50 and older reported the mediating role of income on the relationship between education and cognitive function (30).

In contrast to earlier studies (1, 9), we failed to observe any associations between alcohol drinking, unfavourable sleep duration, and cognitive function. This could be attributed to the oversimplified categorization of non-drinkers and drinkers, as well as the possibly inappropriate definition of unfavourable sleep duration for the Chinese population, which was adopted from a UK study (14). Consistent with our study, a cross-sectional study of 4,631 Chinese adults aged 60 and older also found an association between higher BMI and reduced MCI incidence (12). Overweight and obesity in later life may lead to increased secretion of leptin hormone from adipose tissue, which could reduce deposition of amyloid-*β* (Aβ) in the brain, thereby lower the risk of cognitive impairment and AD (33). Furthermore, the observed protective effect of obesity on cognitive function may be related to the robust link between elevated BMI and increased hippocampal volumes, as larger hippocampal volumes are often associated with better cognitive function (34). When the sample was stratified by sex and age, the impact of smoking was only evident in men and those aged 70 and older, which may be due to the sexed pattern of smoking and the time required for smoking as an accumulated exposure to affect cognitive function (35). In addition, unfavourable sleep duration only exhibited an adverse effect on cognitive function in individuals aged 50–59. This could be linked to the activation of low-level systemic inflammation – an important mechanism for cognitive decline – which may affect younger individuals more than older ones (8, 36). The omission of nap time in calculating total sleep duration in our study may overlook the compensatory effects of napping on nighttime sleep.

The associations between stroke, probable depression, and cognitive function were robust across sexes, age groups, and rural–urban residences. The biological mechanisms that connect stroke to cognitive decline encompass AD-related pathologies triggered or accelerated by stroke, brain injuries exacerbated by pre-stroke neurodegeneration, and vascular-related comorbidities (e.g., hypertension and atrial fibrillation) (37). Whereas for depression, it involved white matter hyperintensities, decreased hippocampal volume, reduction of prefrontal cortical thickness, and elevated levels of *β*-amyloid (38). Nevertheless, contradicting previous findings (5, 8), we found older Chinese adults with heart disease and dyslipidaemia had better cognitive function. Elevated cholesterol levels may be indicative of better nutritional status and general health, particularly in later life (39). Moreover, individuals with dyslipidemia and heart disease may be on long-term statins and antihypertensive medications, which have been shown to be effective in preventing cognitive impairment (40) and dementia (8). Our findings could also be attributed to the simultaneous inclusion of hypertension, obesity, dyslipidemia, diabetes, heart disease, and stroke in the model, which were correlated with each other and thus may distort the relationship between specific health conditions and cognitive function.

The marked sex difference in how the socioeconomic disadvantage risk score affected cognitive function, as observed in our study compared to the UK Biobank study (14), suggests greater health inequalities for women than men in China as opposed to the UK (41). In addition, we found that the effects of socioeconomic disadvantage and lifestyle risk scores increased with age, contrary to the findings from the UK Biobank study (14). This discrepancy may be ascribed to differences in outcome measures—our study centred on cognitive function, while the other study relied on dementia diagnoses extracted from electronic health records, potentially leading to a downward bias in dementia incidence estimation (42). Cognitive deterioration progresses along a continuum from normal cognition to subjective cognitive decline, MCI, and ultimately to dementia. The lack of data on dementia from Waves 1–3 in CHARLS, with the exception of Wave 4, has constrained us to investigate the relationships between modifiable risk factors and dementia. Furthermore, the observed age trends in the UK Biobank study may partially reflect fundamental differences between early- and late-onset dementia, which is less of a concern for our study. Most importantly, our study accounted for mortality selection and non-ignorable missingness caused by dropouts due to death by jointly modelling the longitudinal and survival processes, which the UK Biobank study failed to. Jointly modelling the longitudinal and survival processes enabled us to obtain more efficient and precise estimates of the relationships between the risk scores and trajectories of cognitive function while mitigating bias (43). In our joint models, the associations between modifiable risk factors and cognitive function only changed by less than 5%, suggesting that mortality selection and dropouts due to death might play a negligible role in the Chinese population. This could also be attributed to the same assumptions shared by the separate and joint models, and to the lack of severe censoring of cognitive function due to deaths in our study, as only 10.2% of the participants died during the follow-up period (44).

Consistent with prior research (16, 45), our study showed that the change in cognitive function with age, rather than the initial levels of cognitive function, was associated with a reduced risk of mortality. A cohort study from China reported a stronger association between cognitive impairment and mortality among individuals aged 65 and above compared to those under 65 (46), but a study from the UK demonstrated the opposite (15). We found that the magnitude of the cognition-mortality association rose with age, which could be partly attributed to the less prevalent cognitive decline in younger-old adults that may hold greater clinical significance, be more indicative of underlying brain disease of any etiology, and be more closely linked with underlying diseases that elevate mortality risk (16, 45). However, it has been well-recognized that dropouts due to death may also play an important role, as we are not only unable to continue following individuals who have died, but it is also possible that at advanced ages, the survivors may successfully adapt to mild cognitive deficits (47). This could explain the age pattern we observed after factoring in dropouts due to death.

We acknowledge that our study has several strengths and limitations. Utilizing longitudinal data from CHARLS with a nationally representative sample maximized the generalization of our findings to the middle-aged and older population of China. To our knowledge, this is the first study to jointly model trajectories of cognitive function and mortality when examining the associations between modifiable risk factors and cognitive function, which enabled us to account for mortality selection and non-ignorable missingness caused by dropouts due to death (23). We estimated the joint models using the Bayesian approach with MCMC posterior simulations, which has been demonstrated to outperform the frequentist approach based on maximum likelihood in terms of bias, flexibility, and coverage (24, 48). However, generalizing our findings to other populations should be done with caution due to possibly large differences in the distributions of cognitive function, modifiable risk factors, and covariates, as well as their associations with mortality. It is possible that socioeconomic characteristics and lifestyle can influence cognitive function and mortality through triggering hypertension, dyslipidaemia, and CVD (49). Future research is needed to examine these mediating effects while taking mortality selection (e.g., mortality due to CVD) into consideration.

## Conclusion

Mortality selection and non-ignorable missingness caused by dropouts due to death played a minor role in the associations between modifiable risk factors and cognitive function in middle-aged and older Chinese adults. The large sex difference and the age trend underscore that, to maintain good cognitive function and prevent cognitive impairment and dementia, it is necessary to address socioeconomic inequalities between sexes and target individuals at older ages with socioeconomic disadvantage and an unfavourable lifestyle.

## Data Availability

Publicly available datasets were analyzed in this study. This data can be found here: http://charls.pku.edu.cn/.

## Footnotes

^1^ https://charls.pku.edu.cn/en/

## References

[1] Livingston G Huntley J Sommerlad A Ames D Ballard C Banerjee S. Dementia prevention, intervention, and care: 2020 report of the lancet commission. Lancet. (2020) 396:413–46. doi: 10.1016/S0140-6736(20)30367-6, PMID: 32738937 PMC7392084

[2] Manly JJ Jones RN Langa KM Ryan LH Levine DA McCammon R. Estimating the prevalence of dementia and mild cognitive impairment in the US: the 2016 health and retirement study harmonized cognitive assessment protocol project. JAMA Neurol. (2022) 79:1242–9. doi: 10.1001/jamaneurol.2022.3543, PMID: 36279130 PMC9593315

[3] Kim YJ Han JW So YS Seo JY Kim KY Kim KW. Prevalence and trends of dementia in Korea: a systematic review and meta-analysis. J Korean Med Sci. (2014) 29:903–12. doi: 10.3346/jkms.2014.29.7.903 25045221 PMC4101777

[4] Shimizu H Mori T Yoshida T Tachibana A Ozaki T Yoshino Y. Secular trends in the prevalence of dementia based on a community-based complete enumeration in Japan: the Nakayama study. Psychogeriatrics. (2022) 22:631–41. doi: 10.1111/psyg.12865, PMID: 35753054 PMC9541546

[5] Jia L Du Y Chu L Zhang Z Li F Lyu D. Prevalence, risk factors, and management of dementia and mild cognitive impairment in adults aged 60 years or older in China: a cross-sectional study. Lancet Public Health. (2020) 5:e661–71. doi: 10.1016/S2468-2667(20)30185-7 33271079

[6] Livingston G Sommerlad A Orgeta V Costafreda SG Huntley J Ames D. Dementia prevention, intervention, and care. Lancet. (2017) 390:2673–734. doi: 10.1016/S0140-6736(17)31363-6 28735855

[7] Kivipelto M Mangialasche F Ngandu T. Lifestyle interventions to prevent cognitive impairment, dementia and Alzheimer disease. Nat Rev Neurol. (2018) 14:653–66. doi: 10.1038/s41582-018-0070-3 30291317

[8] Zhang Y Xu W Zhang W Wang H-F Ou Y-N Qu Y. Modifiable risk factors for incident dementia and cognitive impairment: An umbrella review of evidence. J Affect Disord. (2021) 314:160–7. doi: 10.1016/j.jad.2022.07.008, PMID: 35863541

[9] Ma Y Liang L Zheng F Shi L Zhong B Xie W. Association between sleep duration and cognitive decline. JAMA Netw Open. (2020) 3:e2013573. doi: 10.1001/jamanetworkopen.2020.13573, PMID: 32955572 PMC7506513

[10] Guo S Zheng X-Y. New evidence of trends in cognitive function among middle-aged and older adults in China, 2011-2018: an age-period-cohort analysis. BMC Geriatr. (2023) 23:23. doi: 10.1186/s12877-023-04166-9, PMID: 37605117 PMC10440902

[11] Xiang Y Zare H Guan C Gaskin D. The impact of rural-urban community settings on cognitive decline: results from a nationally-representative sample of seniors in China. BMC Geriatr. (2018) 18:323. doi: 10.1186/s12877-018-1003-0, PMID: 30594142 PMC6311043

[12] Fu J Liu Q Du Y Zhu Y Sun C Lin H. Age- and sex-specific prevalence and modifiable risk factors of mild cognitive impairment among older adults in China: a population-based observational study. Front Aging Neurosci. (2020) 12:12. doi: 10.3389/fnagi.2020.578742, PMID: 33192471 PMC7662098

[13] Hu FF Cheng GR Liu D Liu Q Gan XG Li L. Population-attributable fractions of risk factors for all-cause dementia in China rural and urban areas: a cross-sectional study. J Neurol. (2022) 269:3147–58. doi: 10.1007/s00415-021-10886-y, PMID: 34839456

[14] Chen H Cao Y Ma Y Xu W Zong G Yuan C. Age- and sex-specific modifiable risk factor profiles of dementia: evidence from the UK biobank. Eur J Epidemiol. (2023) 38:83–93. doi: 10.1007/s10654-022-00952-8, PMID: 36593335

[15] Hayat SA Luben R Dalzell N Moore S Hogervorst E Matthews FE. Understanding the relationship between cognition and death: a within cohort examination of cognitive measures and mortality. Eur J Epidemiol. (2018) 33:1049–62. doi: 10.1007/s10654-018-0439-z, PMID: 30203336 PMC6208995

[16] Lv X Li W Ma Y Chen H Zeng Y Yu X. Cognitive decline and mortality among community-dwelling Chinese older people. BMC Med. (2019) 17:63. doi: 10.1186/s12916-019-1295-8, PMID: 30871536 PMC6419492

[17] Aichele S Cekic S Rabbitt P Ghisletta P. Cognition-mortality associations are more pronounced when estimated jointly in longitudinal and time-to-event models. Front Psychol. (2021) 12:708361. doi: 10.3389/fpsyg.2021.708361, PMID: 34421759 PMC8378533

[18] Zhao Y Hu Y Smith JP Strauss J Yang G. Cohort profile: the China health and retirement longitudinal study (CHARLS). Int J Epidemiol. (2014) 43:61–8. doi: 10.1093/ije/dys203, PMID: 23243115 PMC3937970

[19] Lei X Hu Y McArdle JJ Smith JP Zhao Y. Gender differences in cognition among older adults in China. J Hum Resour. (2012) 47:951–71. doi: 10.3368/jhr.47.4.951, PMID: 24347682 PMC3859455

[20] Wang J Lu Z Kou S Xie K Wang W Zheng W. Association between obesity and death risk among Chinese adults aged 45 and over (in Chinese). Chin Prev Med. (2022) 23:577–81. doi: 10.16506/j.1009-6639.2022.08.003

[21] Hu Y Ruiz M Bobak M Martikainen P. Four-year trajectories of episodic memory decline in mid-late life by living arrangements: a cross-national comparison between China and England. J Epidemiol Community Health. (2021) 75:881–9. doi: 10.1136/jech-2020-215567, PMID: 33563730

[22] Rizopoulos D. Joint models for longitudinal and time-to-event data: With applications in R. 1st ed. New York: Chapman & Hall/CRC Biostatistics Series (2012).

[23] Chesnaye NC Tripepi G Dekker FW Zoccali C Zwinderman AH Jager KJ. An introduction to joint models—applications in nephrology. Clin Kidney J. (2020) 13:143–9. doi: 10.1093/ckj/sfaa024, PMID: 32296517 PMC7147305

[24] Li K Luo S. Bayesian functional joint models for multivariate longitudinal and time-to-event data. Comput Stat Data Anal. (2019) 129:14–29. doi: 10.1016/j.csda.2018.07.015, PMID: 30559575 PMC6294314

[25] Thiébaut ACM Bénichou J. Choice of time-scale in Cox's model analysis of epidemiologic cohort data: a simulation study. Stat Med. (2004) 23:3803–20. doi: 10.1002/sim.2098, PMID: 15580597

[26] Cekic S Aichele S Brandmaier AM Köhncke Y Ghisletta P. A tutorial for joint modeling of longitudinal and time-to-event data in R. Quant Comput Methods Behav Sci. (2021) 1:e2979. doi: 10.5964/qcmb.2979

[27] R Core Team. R: A language and environment for statistical computing. R Foundation for Statistical Computing, Vienna, Austria. (2022). Available at: https://www.R-project.org/

[28] Payne CF Xu KQ. Life course socioeconomic status and healthy longevity in China. Demography. (2022) 59:629–52. doi: 10.1215/00703370-9830687, PMID: 35292811

[29] Chen Y. Childhood and adult socioeconomic status influence on late-life healthy longevity: evidence from the Chinese longitudinal healthy longevity survey. Front Public Health. (2024) 12:1352937. doi: 10.3389/fpubh.2024.1352937, PMID: 39403433 PMC11471603

[30] Rodriguez FS Hofbauer LM Röhr S. The role of education and income for cognitive functioning in old age: a cross-country comparison. Int J Geriatr Psychiatry. (2021) 36:1908–21. doi: 10.1002/gps.5613 34378818

[31] Lövdén M Fratiglioni L Glymour MM Lindenberger U Tucker-Drob EM. Education and cognitive functioning across the life span. Psychol Sci Public Interest. (2020) 21:6–41. doi: 10.1177/1529100620920576 32772803 PMC7425377

[32] Fujishiro K MacDonald LA Crowe M McClure LA Howard VJ Wadley VG. The role of occupation in explaining cognitive functioning in later life: education and occupational complexity in a U.S. National Sample of black and white men and women. J Gerontol B Psychol Sci Soc Sci. (2019) 74:1189–99. doi: 10.1093/geronb/gbx112, PMID: 28958077 PMC6748817

[33] Lilamand M Bouaziz-Amar E Dumurgier J Cognat E Hourregue C Mouton-Liger F. Plasma leptin is associated with amyloid CSF biomarkers and Alzheimer's disease diagnosis in cognitively impaired patients. J Gerontol A Biol Sci Med Sci. (2023) 78:645–52. doi: 10.1093/gerona/glac234, PMID: 36441007

[34] Sun ZWZ Sun FR Shen XN Xu W Ma YH Dong Q. Late-life obesity is a protective factor for prodromal Alzheimer's disease: a longitudinal study. Aging (Albany NY). (2020) 12:2005–17. doi: 10.18632/aging.102738, PMID: 31986486 PMC7053604

[35] Rusanen M Kivipelto M Quesenberry CP Zhou J Whitmer RA. Heavy smoking in midlife and long-term risk of Alzheimer disease and vascular dementia. Arch Intern Med. (2011) 171:333. doi: 10.1001/archinternmed.2010.393 20975015

[36] Legdeur N Heymans MW Comijs HC Huisman M Maier AB Visser PJ. Age dependency of risk factors for cognitive decline. BMC Geriatr. (2018) 18:18. doi: 10.1186/s12877-018-0876-2 30126373 PMC6102935

[37] Zlokovic BV Gottesman RF Bernstein KE Seshadri S McKee A Snyder H. Vascular contributions to cognitive impairment and dementia (VCID): a report from the 2018 National Heart, Lung, and Blood Institute and National Institute of Neurological Disorders and Stroke workshop. Alzheimers Dement. (2020) 16:1714–33. doi: 10.1002/alz.12157, PMID: 33030307

[38] Jamieson A Goodwill AM Termine M Campbell S Szoeke C. Depression related cerebral pathology and its relationship with cognitive functioning: a systematic review. J Affect Disord. (2019) 250:410–8. doi: 10.1016/j.jad.2019.03.042, PMID: 30878653

[39] Mielke MMZP Sjögren M Gustafson D Ostling S Steen B Skoog I. High total cholesterol levels in late life associated with a reduced risk of dementia. Neurology. (2005) 64:1689–95. doi: 10.1212/01.WNL.0000161870.78572.A5, PMID: 15911792

[40] Sörös P Whitehead S Spence JD Hachinski V. Antihypertensive treatment can prevent stroke and cognitive decline. Nat Rev Neurol. (2012) 9:174–8. doi: 10.1038/nrneurol.2012.255 23247612

[41] Larnyo E Dai B Nutakor JA Ampon-Wireko S Larnyo A Appiah R. Examining the impact of socioeconomic status, demographic characteristics, lifestyle and other risk factors on adults' cognitive functioning in developing countries: an analysis of five selected WHO SAGE wave 1 countries. Int J Equity Health. (2022) 21:21. doi: 10.1186/s12939-022-01622-7, PMID: 35216605 PMC8876754

[42] Mizanur Khondoker AM Bachmann MO Hornberger M Fox C Shepstone L. Multimorbidity pattern and risk of dementia in later life: an 11-year follow-up study using a large community cohort and linked electronic health records. J Epidemiol Community Health. (2023) 77:285–92. doi: 10.1136/jech-2022-220034, PMID: 36889910

[43] Ibrahim JG Chu H Chen LM. Basic concepts and methods for joint models of longitudinal and survival data. J Clin Oncol. (2010) 28:2796–801. doi: 10.1200/JCO.2009.25.0654, PMID: 20439643 PMC4503792

[44] Asar O Ritchie J Kalra PA Diggle PJ. Joint modelling of repeated measurement and time-to-event data: an introductory tutorial. Int J Epidemiol. (2015) 44:334–44. doi: 10.1093/ije/dyu262 25604450

[45] Lavery LL Dodge HH Snitz B Ganguli M. Cognitive decline and mortality in a community-based cohort: the Monongahela Valley independent elders survey. J Am Geriatr Soc. (2009) 57:94–100. doi: 10.1111/j.1532-5415.2008.02052.x, PMID: 19016932 PMC2768614

[46] An J Li H Tang Z Zheng D Guo J Liu Y. Cognitive impairment and risk of all-cause and cardiovascular disease mortality over 20-year follow-up: results from the BLSA. J Am Heart Assoc. (2018) 7:e008252. doi: 10.1161/jaha.117.008252 PMC6201447 30371231

[47] Bassuk SS Wypij D Berkman LF. Cognitive impairment and mortality in the community-dwelling elderly. Am J Epidemiol. (2000) 151:676–88. doi: 10.1093/oxfordjournals.aje.a010262 10752795

[48] Alsefri M Sudell M Garcia-Finana M Kolamunnage-Dona R. Bayesian joint modelling of longitudinal and time to event data: a methodological review. BMC Med Res Methodol. (2020) 20:94. doi: 10.1186/s12874-020-00976-2, PMID: 32336264 PMC7183597

[49] Wright RS Ford C Sniscak CR. Older adult awareness of the influence of cardiovascular disease risk factors on cognitive function. Int J Older People Nursing. (2017) 12:12. doi: 10.1111/opn.12123 27297254

