# Supplementary material for: Associations between modifiable risk factors and cognitive function in middle-aged and older Chinese adults: joint modelling of longitudinal and survival data
Source: Front Public Health. 2024 Nov 18;12:1485556. doi: 10.3389/fpubh.2024.1485556 (PMC11609063; doi:10.3389/fpubh.2024.1485556)
Supplement: Supplementary file 1 [file Data_Sheet_1.PDF]

Supplementary Material

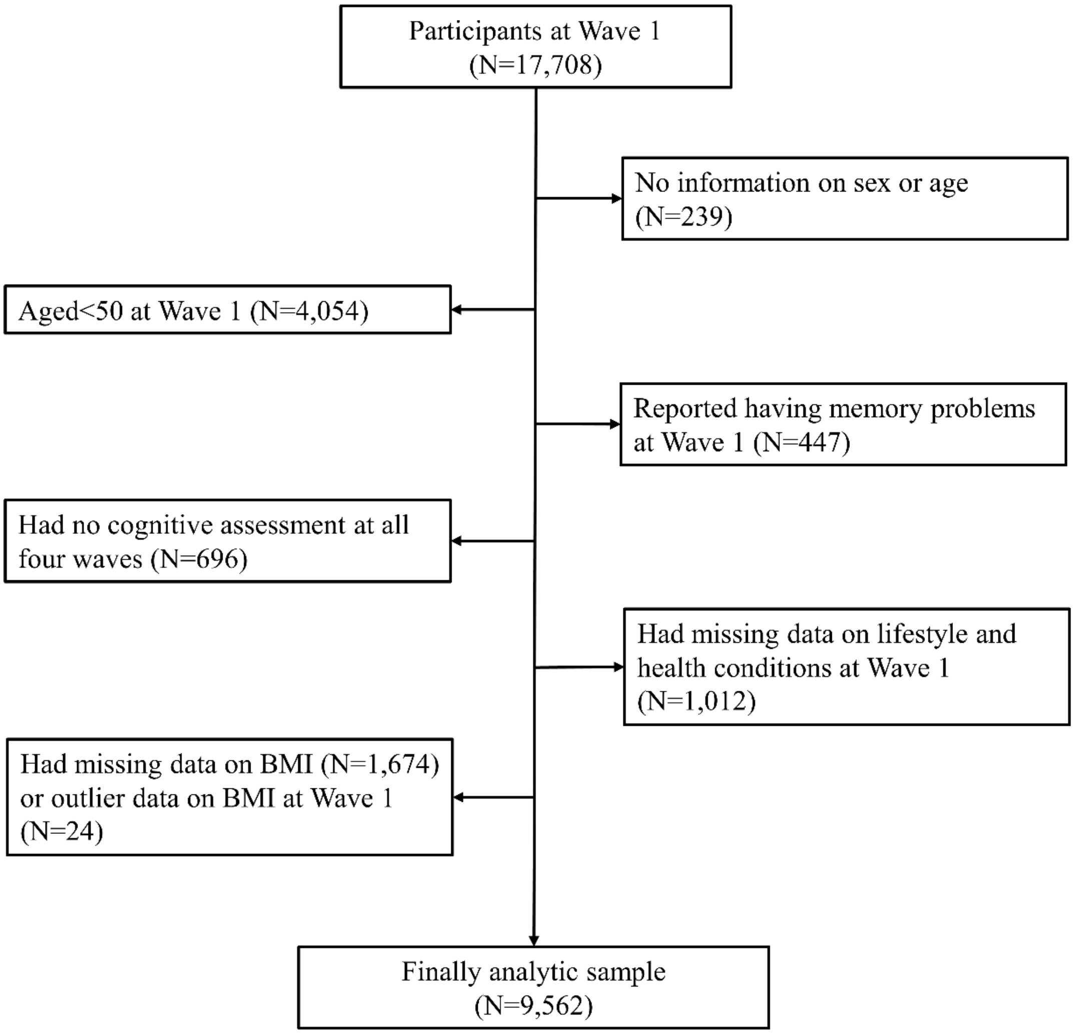

Supplementary Figure 1. Flowchart of the analytical sample.

Supplementary Table 1. Coding of variables

**Variables**	**Coding**
Cognitive function	Continuous variable; range from 0-21
Survival status	Survival=0; death=1
Age	Centered at 50 and then divided by 10
Sex	Men=1; women=2
Marital status	Married/partnered=1; unmarried/divorced/widowed=2
BMI	<24 kg/m^2=1; 24-27.9 kg/m^2=2; ≥28 kg/m^2=3
Socioeconomic disadvantage risk score	Sum of two socioeconomic risk factors (0-2)
Education	≥secondary school=0; <secondary school=1
Place of residence	Residing in rural areas=0; residing in rural areas=1
Health condition risk score	Sum of six health condition risk factors (0-6)
Hypertension	No=0; yes=1
Diabetes	No=0; yes=1
Dyslipidemia	No=0; yes=1
Heart disease	No=0; yes=1
Stroke	No=0; yes=1
Probable depression	CES-D-10<12=0; CES-D-10≥12=1
Lifestyle risk score	Sum of three lifestyle risk factors (0-3)
Smoking	Never/former smoking=0; current smoking=1
Alcohol drinking in the past year	No=0; yes=1
Sleep duration	Age 50-64: 6-10 hours/night=0; ≤6 or ≥10 hours/night=1<br>Age≥65: 6-9 hours/night=0; ≤6 or ≥9 hours/night=1

Supplementary Table 2. Comparisons of linear mixed-effects models

	**AIC**	**BIC**	**Log-likelihood**	**Log-likelihood ratio test[a]**
Random intercept only	142568.7	142733.7	-71264.4	
Random intercept & random slope of age	142448.7	142630.1	-71202.3	<0.001
Random intercept & random slope of age and age squared	142454.3	162660.6	-71202.2	0.954

AIC: Akaike information criterion.
BIC: Bayesian information criterion.
[a] Compare with previous model.

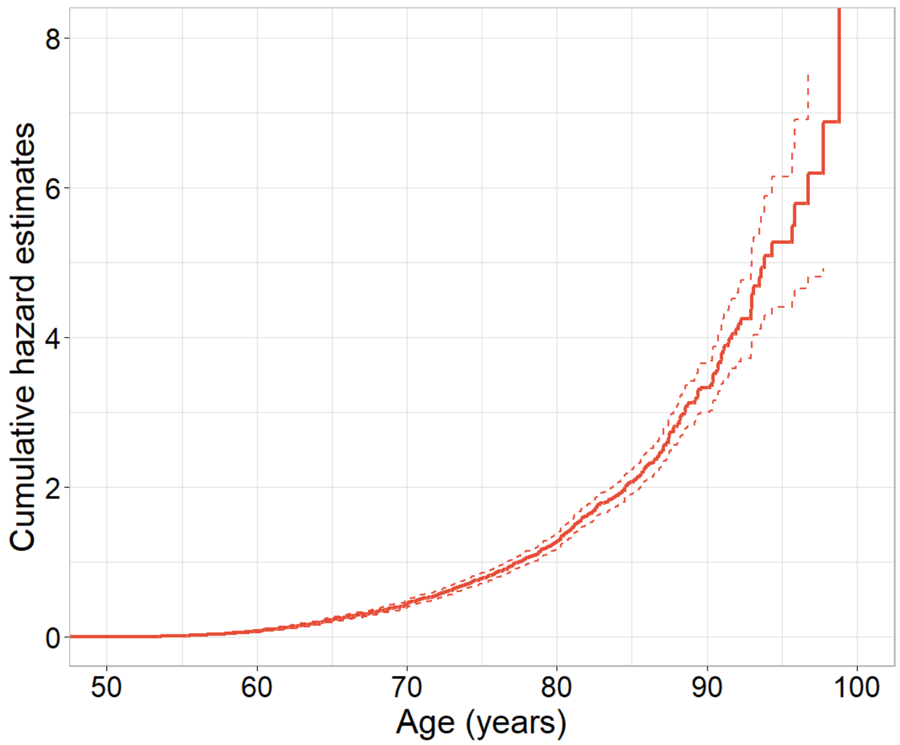

A) Cumulative hazard estimates of mortality using Kaplan-Meier method

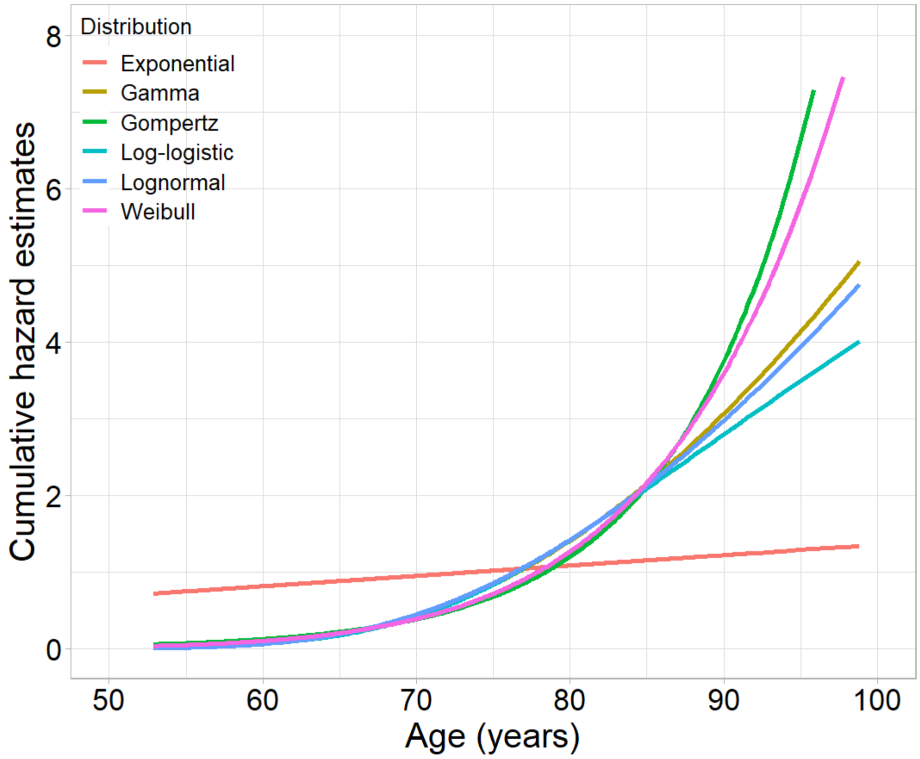

B) Cumulative hazard function of mortality in parametric survival models

Supplementary Figure 2. Cumulative hazard estimates of mortality using Kaplan-Meier method and in parametric survival model

Supplementary Table 3. Comparison of parametric survival models with different survival distributions

Survival distribution	AIC
Weibull	7060
Exponential	8129
Gompertz	7103
Gamma	7145
Log-logistic	7237
Lognormal	7260

AIC: Akaike information criterion

Supplementary Method.

Joint Models can be specified with the following association structures (1):

1) "Current value" association – standard joint model: the hazard of an event at *t* is associated with the current value of the longitudinal outcome at *t*.

$$h_i\big(t|M_i(t)\big) = h_0(t)exp\{\gamma^T\omega_i + \alpha m_i(t)\}$$

$$y_i(t) = m_i(t) + \varepsilon_i(t)$$

$$m_i(t) = x_i^T(t)\beta + z_i^T(t)b_i + \varepsilon_i(t)$$

Where $M_i(t) = \{m_i(s), 0 \leq s < t\}$ denotes the history of the true unobserved longitudinal process up to time point *t*, $h_0(t)$ denotes the baseline hazard, ω_i indicates baseline covariates with a corresponding vector of regression coefficients γ. Besides, α quantifies the association between the longitudinal outcome and the risk of an event.

$y_i(t)$ denote the observed longitudinal data for the *i*th subject at time *t*, $m_i(t)$ denotes the true & unobserved value of the longitudinal data at time *t*, β is fixed-effects regression coefficients, b_i is random-effects regression coefficients, $x_i(t)$ and $z_i(t)$ denote the design vectors for fixed-effects and random-effects respectively. $\varepsilon_i(t)$ indicates the measurement error term, which we assume that it is independent of the random effects and follows normally distributed with mean zero and variance σ^2.

2) "Current slope" association: the hazard of an event at *t* is only associated with the slope of

the trajectory of the longitudinal outcome at *t*.

$$h_i(t|M_i(t)) = h_0(t)exp\{\gamma^T\omega_i + \alpha_2 m_i'(t)\}$$

$$m_i'(t) = \frac{d}{dt}\{x_i^T(t)\beta + z_i^T(t)b_i\}$$

Where α_2 denotes the association between the slope value of the true longitudinal trajectory at time *t* and the risk for event at the same time point.

3) "Current value and current slope" association: the hazard of an event at *t* is associated with both the current value and the slope of the trajectory of the longitudinal outcome at *t*.

$$h_i(t|M_i(t)) = h_0(t)exp\{\gamma^T\omega_i + \alpha_1 m_i(t) + \alpha_2 m_i'(t)\}$$

$$m_i'(t) = \frac{d}{dt}\{x_i^T(t)\beta + z_i^T(t)b_i\}$$

Where α_1 remains the same with α in the standard parameterization.

4) "Time-dependent slope" association: the hazard of an event at *t* is associated with the change of the longitudinal outcome between *t-1* and *t*.

$$h_i(t|M_i(t)) = h_0 exp\{\gamma^T\omega_i + \alpha\Delta m_i(t)\}$$

$$\Delta m_i(t) = m_i(t) - m_i(t-1)$$

Reference:

1. Rizopoulos, D. (2012). Joint Models for Longitudinal and Time-to-Event Data: With Applications in R, 1st ed. Chapman & Hall/CRC, https://doi.org/10.1201/b12208.

Supplementary Table 4. Comparison of joint models with different association structures

**Association structure**	**DIC**	**WAIC**	**LPML**
Current value	150737.0	150785.5	-75392.8
Current slope	150760.2	150783.6	-75391.8
Current value and current slope	150589.3	150758.6	-75379.3
Time-dependent slope	150735.7	150765.5	-75383.0

DIC: deviance information criterion.
LPML: logarithm of the pseudo marginal likelihood.
WAIC: Watanabe-Akaike information criterion.

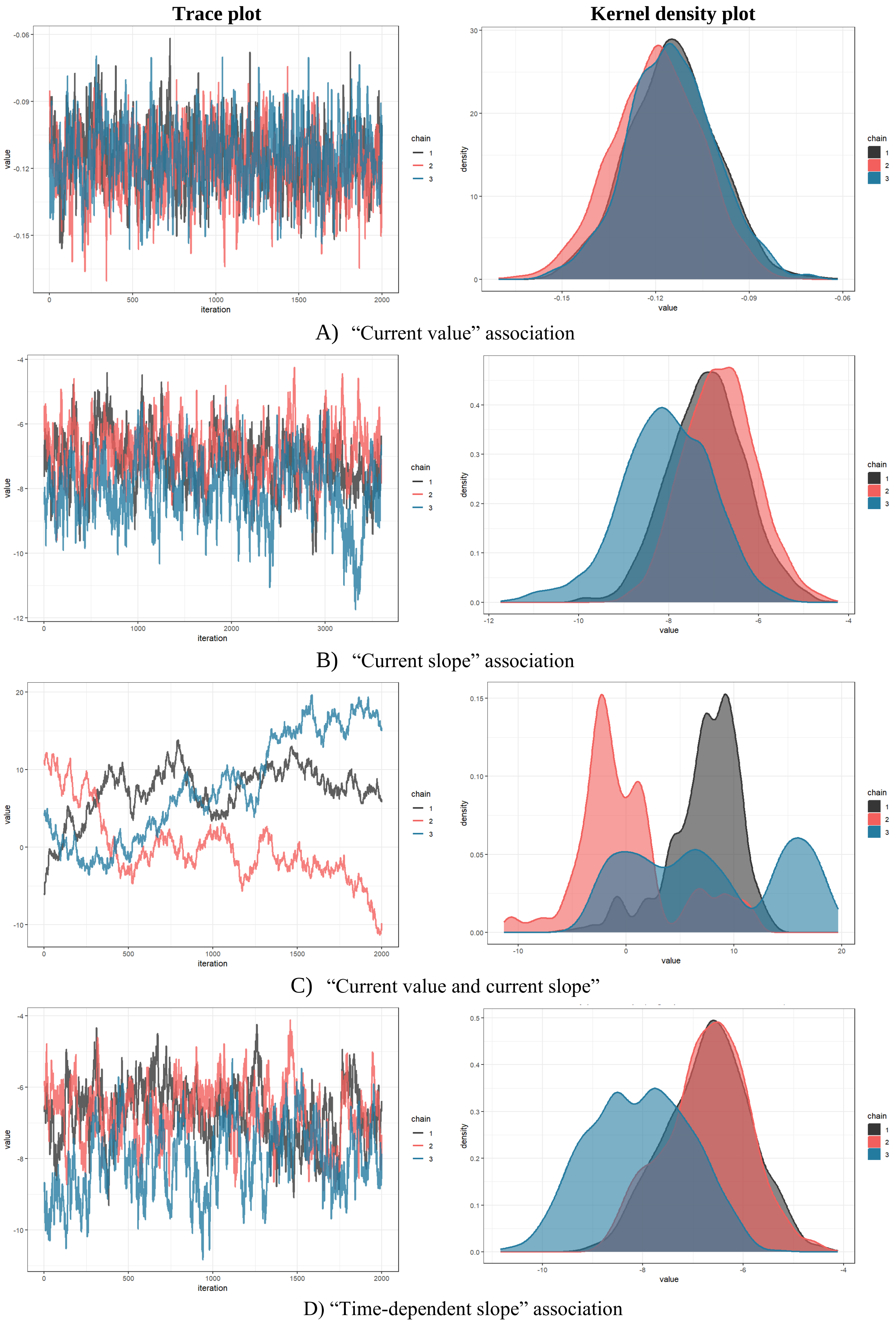

Supplementary Figure 3. Diagnostic plots of joint models with different association structures

Supplementary Table 5. Associations of risk scores for socioeconomic disadvantage, lifestyle, and health conditions with cognitive function and mortality by sex and age group

	**Total sample**		**Men**		**Women**		**50-59 years**		**60-69 years**		**≥70 years**	
	**β/HR (95% CI)**	**P**	**β/HR (95% CI)**	**P**	**β/HR (95% CI)**	**P**	**β/HR (95% CI)**	**P**	**β/HR (95% CI)**	**P**	**β/HR (95% CI)**	**P**
**Longitudinal process**												
Age	-0.004 (-0.243, 0.195)	0.993	0.242 (-0.088, 0.728)	0.302	-0.285 (-0.520, 0.030)	0.137	0.472 (0.084, 0.862)	0.010*	-0.678 (-1.125, -0.218)	0.001**	-2.236 (-2.983, -1.199)	<0.001***
Age squared	-0.494 (-0.559, -0.423)	<0.001***	-0.509 (-0.656, -0.403)	<0.001***	-0.465 (-0.569, -0.383)	<0.001***	-1.151 (-1.378, -0.931)	<0.001***	-1.017 (-1.300, -0.725)	<0.001***	-0.698 (-1.356, -0.104)	0.006**
Women	-1.913 (-2.059, -1.767)	<0.001***					-1.416 (-1.633, -1.203)	<0.001***	-2.082 (-2.324, -1.837)	<0.001***	-2.705 (-3.068, -2.345)	<0.001***
Socioeconomic disadvantage	-2.064 (-2.152, -1.977)	<0.001***	-1.597 (-1.709, -1.485)	<0.001***	-2.572 (-2.710, -2.436)	<0.001***	-2.078 (-2.194, -1.959)	<0.001***	-2.111 (-2.264, -1.957)	<0.001***	-2.247 (-2.499, -1.997)	<0.001***
Health conditions	-0.043 (-0.106, 0.021)	0.192	-0.055 (-0.140, 0.032)	0.218	-0.031 (-0.121, 0.060)	0.512	-0.125 (-0.216, -0.032)	0.009**	-0.039 (-0.138, 0.063)	0.443	-0.162 (-0.332, 0.006)	0.059
Lifestyle	-0.230 (-0.308, -0.150)	<0.001***	-0.219 (-0.312, -0.130)	<0.001***	-0.210 (-0.358, -0.061)	0.006**	-0.177 (-0.289, -0.063)	0.001**	-0.129 (-0.264, 0.008)	0.063	-0.435 (-0.645, -0.225)	<0.001***
**Survival process**												
Women	0.480 (0.397, 0.585)	<0.001***					0.384 (0.240, 0.614)	<0.001***	0.425 (0.309, 0.587)	<0.001***	0.285 (0.110, 0.611)	<0.001***
Socioeconomic disadvantage	1.054 (0.949, 1.173)	0.329	1.034 (0.887, 1.208)	0.685	1.081 (0.852, 1.379)	0.543	0.937 (0.756, 1.163)	0.559	1.128 (0.952, 1.336)	0.156	1.509 (1.040, 2.436)	0.028*
Health conditions	1.214 (1.144, 1.286)	<0.001***	1.166 (1.070, 1.264) ***	<0.001***	1.293 (1.165, 1.429)	<0.001***	1.272 (1.103, 1.466)	0.001**	1.314 (1.198, 1.439)	<0.001***	1.499 (1.166, 2.172)	<0.001***
Lifestyle	1.022 (0.941, 1.110)	0.602	1.023 (0.936, 1.116)	0.609	0.999 (0.846, 1.179)	0.997	1.064 (0.884, 1.289)	0.525	1.012 (0.886, 1.158)	0.565	0.912 (0.664, 1.187)	0.501
Time-dependent slope	0.411 (0.327, 0.504)	<0.001***	0.531 (0.387, 0.668)	<0.001***	0.298 (0.180, 0.488)	<0.001***	0.682 (0.527, 0.849)	<0.001***	0.673 (0.561, 0.792)	<0.001***	0.082 (0.023, 0.402)	<0.001***

β: model coefficient; CI: confidence interval; HR: hazard ratio.
*P <.05, **P <.01, ***P <.001
